# Distinct Roles of apoE Receptor-2 Cytoplasmic Domain Splice Variants in Cardiometabolic Disease Modulation

**DOI:** 10.3390/biomedicines13071692

**Published:** 2025-07-10

**Authors:** Anja Jaeschke, April Haller, David Y. Hui

**Affiliations:** Department of Pathology and Laboratory Medicine, University of Cincinnati College of Medicine, Cincinnati, OH 45237, USA; anjajaeschke227@gmail.com (A.J.); cannonam@ucmail.uc.edu (A.H.)

**Keywords:** LDL receptor-related proteins, alternative splicing/genetics, atherosclerosis, neointima formation, hyperglycemia

## Abstract

**Background/Objectives**: Apolipoprotein E receptor-2 (apoER2) exists in various alternatively spliced forms, including variants that express apoER2 with or without exon 19 in the cytoplasmic domain. This study compared vascular response to endothelial denudation, as well as diet-induced atherosclerotic and metabolic diseases, between genetically modified mice that exclusively expressed the apoER2 splice variant with or without exon 19 to determine the impact of apoER2 exon 19 motif in cardiometabolic disease modulation. **Methods**: Vascular response to injury was assessed by measuring neointima area of the carotid arteries after endothelial denudation. The genetically modified mice were also fed a high-fat high-cholesterol diet for 16 weeks for the determination of body weight gain, glucose and insulin levels, glucose tolerance and insulin secretion. Additionally, adipose tissue inflammation was assessed by analysis of adipose gene expression, and atherosclerosis was characterized by measuring fatty lesion size in the whole aorta, as well as in the aortic roots. **Results**: The results showed that whereas the expression of either splice variant is sufficient to impede denudation-induced fibrotic neointima formation and complex necrotic atherosclerotic lesions, the expression of the apoER2 splice variant containing exon 19 is necessary for the complete protection of injury-induced neointima formation in the vessel wall. However, exclusive expression of either apoER2 cytoplasmic splice variant does not influence the early phase of atherogenesis. Additionally, the exclusive expression of apoER2 without exon 19 promotes adipocyte inflammation and accelerates diet-induced insulin resistance and glucose intolerance. **Conclusions**: These results indicate that the apoER2 cytoplasmic variants have distinct and cell type-specific roles in influencing cardiometabolic disease development.

## 1. Introduction

Apolipoprotein E receptor 2 (apoER2), encoded by the *LRP8* gene, is a single transmembrane protein with structural features similar to those of other members in the LDL receptor family. The extracellular domain of these proteins includes a ligand binding domain consisting multiple cysteine-rich repeats of 40 residues, an epidermal growth factor precursor homology domain and an O-linked glycosylation domain immediately upstream of the membrane-spanning domain. The intracellular domain downstream of the membrane-spanning sequence includes motifs that interact with intracellular adaptor proteins. Ligand binding to the extracellular domain of these receptors results in their endocytosis and lysosomal degradation and/or signal transmission to the cell interior to regulate cell functions. The endocytic versus signal transduction functions of these receptors are dependent on the intracellular domain interaction with specific adaptor proteins in the cell interior. A common intracellular motif shared by all members in the LDL receptor family is the NPxY motif that can interact with both endocytic adaptor proteins and signal transduction adaptor proteins. Interestingly, although apoER2 also contains a single NPxY motif and its interaction with the adaptor protein disabled-2 can mediate endocytosis [1], apoER2 is primarily a signal transduction receptor with a slow endocytosis rate [2]. The preference of apoER2 for signal transduction purposes is apparently due to the presence of a unique alternatively spliced exon (murine exon 19, human exon 18) encoding a 59-amino acid residue insert containing a PxxP motif that excludes apoER2 from clathrin-coated pits and prevents it from causing clathrin-mediated endocytosis [3]. This proline-rich cytoplasmic insert also interacts with numerous adaptor proteins and signaling molecules, such as Src homology domain-containing transforming protein 1, protein phosphatase 2A (PP2A) [4], the c-jun NH_2_-terminal kinase (JNK)-interacting proteins 1 and 2 (JIP1, JIP2) [5] and postsynaptic density protein-95 [6]. The interaction of these signaling molecules with the unique cytoplasmic insert indicates the importance of this alternatively spliced exon in the regulation of specific cell functions by apoER2.

The role of the apoER2 cytoplasmic insert in the selective modulation of cell functions was evident in studies comparing synaptic plasticity, brain development and memory observed with homologous knockin gene replacement mice that exclusively expressed the nonspliceable isoform (*Lrp8^+exon19^*) or the alternatively spliced form missing exon 19 (*Lrp8^Δexon19^*) [7]. Whereas neuronal positioning in the neocortex and the hippocampus during the cerebellum development of *Lrp8^+exon19^* and *Lrp8^Δexon19^* mice was comparable to that observed in wild-type mice, the expression of the nonspliceable apoER2 isoform necessary for long-term potentiation in adult brains and *Lrp8^Δexon19^* mice that expressed only the alternatively spliced form of apoER2 was similar to that for *Lrp8^−/−^* mice with impairment in learning and memory capabilities [7]. These data indicated that the presence of the alternatively spliced exon 19 is not necessary for apoER2-mediated tyrosine kinase activation during brain development, but apoER2-mediated NMDA receptor activation required for long-term potentiation and hippocampus-dependent spatial learning is dependent on the presence of the alternatively spliced exon 19. Similarly, apoER2-mediated selenoprotein P uptake by testis and sperm motility also showed differences in the requirement for exon 19. Whereas apoER2-mediated selenoprotein P endocytosis was not affected in *Lrp8^Δexon19^* mice, sperm motility was impaired in mice expressing only the apoER2 variant without exon 19 [8].

In addition to the central nervous system and testis where apoER2 is abundantly expressed, apoER2 is also expressed in cells and tissues that play a prominent role in modulating the progression of cardiovascular and metabolic diseases. In particular, apoER2 plays an important role in limiting denudation injury-induced neointima formation in the vessel wall [9], and the absence of apoER2 in *Lrp8^−/−^* mice accelerates the hypercholesterolemia-induced progression of atherosclerotic plaques to a more complex necrotic lesion [10]. In contrast, *Lrp8^−/−^* mice fed a high-fat–high-cholesterol Western-type diet displayed lower adiposity with slower development of hyperinsulinemia but accelerated onset of hyperglycemia [11]. Whether exon 19 in apoER2 is necessary for these cardiometabolic disease modulation properties has not been explored. This study examined cardiovascular occlusive diseases and diet-induced metabolic diseases in *Lrp8^+exon19^*, *Lrp8^Δexon19^* and wild-type mice expressing both splice variants to address this issue.

## 2. Materials and Methods

### 2.1. Animal Models

Mutant mice that constitutively express either the nonspliceable variant of apoER2 with the exon 19 cytoplasmic insert (*Lrp8^+exon19^*) or the alternatively spliced form without the exon 19 cytoplasmic insert (*Lrp8^Δexon19^*) were originally generated in the Herz Laboratory at the University of Texas Southwestern Medical Center [7], and they were obtained from the Jackson Laboratory (Bar Harbor, ME, USA). The mice were backcrossed to a C57BL/6J background at our institution vivarium for >10 generations before use. For atherosclerosis studies, mice were also mated with *Ldlr^−/−^* mice (Jackson Laboratory) to generate *Lrp8^WT^Ldlr^−/−^*, *Lrp8^+exon19^Ldlr^−/−^* and *Lrp8^Δexon19^Ldlr^−/−^* mice to assess how apoER2 exon 19 cytoplasmic splice variants may influence atherosclerosis progression. Colonies of wild-type C57BL/6J and *Ldlr^−/−^* mice were also established in our animal facility to obtain wild-type and *Ldlr^−/−^* mice maintained under identical conditions. Wild-type, *Lrp8^+exon19^* and *Lrp8^Δexon19^* mice were fed a standard rodent chow diet (Purina PicoLab Rodent Irradiated Diet 5053; Cincinnati Lab Supply, Cincinnati, OH, USA) or a Western-type diet containing 42% fat and 0.2% cholesterol (TD88137, Envigo Teklad, Madison, MI, USA) for 16 weeks beginning at 12 weeks of age. Mice were euthanized at the end of the experimental period by overdose isoflurane inhalation and the removal of vital organs. Only male mice were used for all experiments to exclude the confounding variable of estrous cycle effects in females and for direct comparison with previously published data on *Lrp8^−/−^* mice [10,11]. All procedures and animal care techniques were approved by the Institutional Animal Care and Use Committee of the University of Cincinnati. This study was carried out in accordance with guidelines and regulations of the National Institutes of Health, USA, and in compliance with ARRIVE guidelines.

### 2.2. Endothelial Denudation of the Carotid Arteries

The endothelial denudation of the carotid arteries was performed as described previously [12]. In brief, a midline neck incision was made and the exposed left external carotid artery was looped with 6-0 silk suture for temporary vascular control. A transverse arteriotomy was made and an epon resin probe with diameter slightly larger than the diameter of the mouse carotid artery was inserted and advanced 5 times at a site immediately distal from the branch point of the external carotid toward the aortic arch. The neointima area was assessed after 14 days by examining decalcified paraffin-embedded cross sections. Ten identical 5 µm intervals were made beginning at the point where the epon resin probe was inserted. Parallel sections were subjected to hematoxylin and eosin staining, as well as to Verhoeff–Van Gieson staining, to identify the elastic lamina. Morphometric analysis was performed with Verhoeff–Van Gieson-stained sections. Four cross sections were digitized, and neointima in the vessels were quantified with Scion Image analysis version 4.0.2 software.

### 2.3. Smooth Muscle Cell Isolation and Culture

Smooth muscle cells were obtained from the thoracic aortas, as described previously [9]. In brief, the thoracic aortas dissected from the mice were incubated in Hanks’ solution containing 1 mg/mL of collagenase and 0.5 mg/mL of elastase for 30 min at 37 °C. Cell clumps were dissociated by aspiration through a 10 mL pipette, and the cell suspension was centrifuged at 150× *g* for 5 min and then resuspended in DMEM with low glucose (Life Technologies, Carlsbad, CA, USA) containing 10% fetal bovine serum (Invitrogen, Carlsbad, CA, USA), 100 U/mL of penicillin 100 µg/mL of streptomycin solution (Thermo Fisher Scientific, Cincinnati, OH, USA) and 2 mmol/L of L-glutamine (Thermo Fisher Scientific) at 37 °C and 5% CO_2_. Growth rates were determined by plating freshly isolated cells at 10,000 cells per well in 12-well cell culture dishes. Cell growth was monitored based on crystal violet staining detected at the optical density of 590 nm.

### 2.4. Body Composition and Plasma Chemistry

Body weights were obtained after 15 weeks of feeding the standard rodent chow or Western diet, and body fat and lean mass were measured in conscious mice using ^1^H magnetic resonance spectroscopy (EchoMRI-100, Echo-Medical Systems, Houston, TX, USA). Blood samples were collected from the tail vein after an overnight fast into EDTA-containing tubes. Plasma was prepared by centrifugation at 1500× *g* for 10 min at 4 °C. Plasma cholesterol and triglyceride levels were quantified by Infinity colorimetric assay kits (TR22421 and TR13421, Thermo Fisher Scientific). Glucose levels were measured using an Accu-Chek glucometer (Roche Applied Sciences, Indianapolis, IN, USA), and insulin levels were determined by the UltraSensitive Mouse Insulin ELISA kit (Crystal Chem, Chicago, IL, USA). A homeostatic model assessment of insulin resistance (HOMA-IR) was calculated using the formula [glucose (mmol/L) × insulin (µU/mL)]/22.5.

### 2.5. Glucose Tolerance and Insulin Secretion Assays

Glucose tolerance and insulin secretion were determined after oral administration of a bolus glucose meal (2 g/kg body weight) to fasting animals. Glucose tolerance was determined by measuring blood glucose levels through a 2 h period. The glucose-induced insulin secretion rate was estimated based on plasma insulin levels measured at 15 and 30 min after glucose feeding, as described in [11].

### 2.6. Gene Expression Analysis

Gene expression was analyzed by quantitative real-time PCR. RNA was isolated from epididymal adipose tissues using TRIzol reagent (Invitrogen), followed by cDNA synthesis using the qScript cDNA Synthesis Kit (QuantaBio, Beverley, MA, USA). Quantitative real-time PCR was performed with a StepOnePlus Thermocycler using Fast SYBR Green Master Mix (Applied Biosystems, Carlsbad, CA, USA). Sequence-specific primers are listed in Table 1. Expression levels of mRNA were normalized to cyclophilin using the ΔΔCT analysis method. The data were reported as a fold change compared to the mean values obtained from wild-type mice.

### 2.7. Atherosclerosis Characterization

Atherosclerosis was characterized by en face analysis of the whole aorta, as well as cryosections of the aortic roots, as described previously [13]. In brief, anesthetized mice were perfused with phosphate-buffered saline, followed by 10% formalin, before harvesting the heart and the entire aorta to the ileac bifurcation. En face analysis of atherosclerotic lesions in the whole aorta was accomplished by opening the aortas longitudinally from the bifurcation of the subclavian and carotid arteries to the iliac bifurcation and then stained with Oil Red O for 30 min. For analysis of the aortic roots, the hearts were fixed in 4% paraformaldehyde and embedded in Scigen Tissue-Plus^TM^ OCT compound (Thermo Fisher Scientific). Cryosections of 5 µm thickness were performed, starting at the valve nubs and the appearance of the coronary artery branch throughout the aortic sinus until the valve separates at the base of the heart. The cryosections were mounted on Premium Superfrost^®^ Plus Microscope Slides (VWR International, Radnor, PA, USA) and then stained with Oil Red O and counterstained with hematoxylin to measure neutral lipid accumulation. All images were taken using an Olympus BX61 microscope and were subsequently quantified using ImageJ software, version 1.52 (NIH).

### 2.8. Statistical Analysis

All data were expressed as mean ± SD. Statistical analysis was performed using GraphPad Prism version 5.0 software (San Diego, CA, USA). Normality was assessed by the Shapiro–Wilk test. Data with normal distribution were then subjected to multiple group comparisons by one-way ANOVA with Student–Newman–Keuls post hoc analysis and Student’s *t* test when evaluating differences between two groups. Data with nonparametric distribution were analyzed by the Kruskal–Wallis test, followed by a Dunn test evaluation of differences for multiple group comparisons, or by the Mann–Whitney test for comparison between 2 groups. Differences at *p* < 0.05 were considered statistically significant.

## 3. Results

### 3.1. Impact of apoER2 Exon 19 in Terms of Modulating Sensitivity to Injury-Induced Neointima Formation

**Figure 1:**
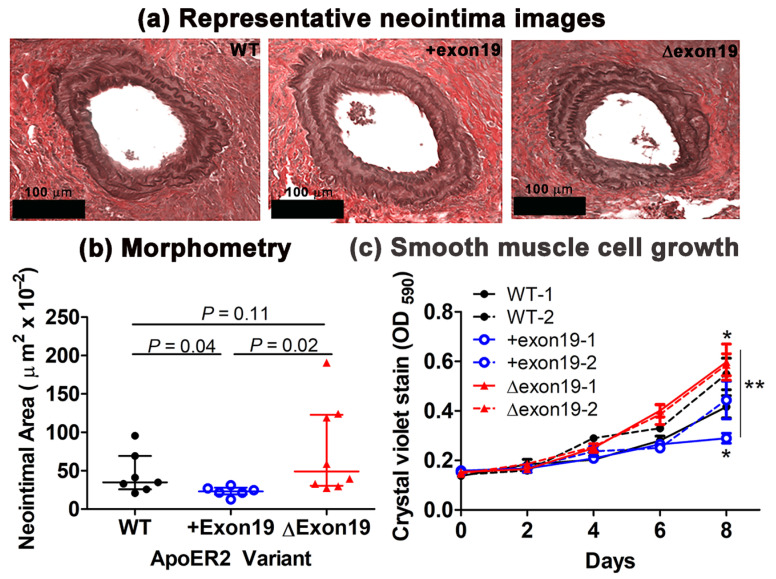
The effect of the apoER2 exon 19 splice variant on denudation-induced neointima formation and smooth muscle cell growth. Wild-type (WT), *Lrp8^+exon19^* and *Lrp8^Δexon19^* mice fed a regular chow diet were subjected to endothelial denudation of the carotid arteries. The arteries were analyzed histologically after 14 days. (**a**) Representative histological images (scale bar = 100 µm) and (**b**) morphometric analysis of data collected from 7 WT mice, 6 *Lrp8^+exon19^* and 8 *Lrp8^Δexon19^* mice. Data were evaluated by the Shapiro–Wilk test for normality and then analyzed by one-way ANOVA with the Kruskal–Wallis post hoc test. The data are presented as median with interquartile range. Differences between groups are shown with *p* values, as indicated. (**c**) Smooth muscle cells isolated from WT, *Lrp8^+exon19^* and *Lrp8^Δexon19^* mice (n = 2 mice in each group) were cultured in triplicates in 10% fetal bovine serum at a density of 10,000 cells in 12-well dishes. Cell growth was monitored by crystal violet staining. The data were analyzed by nonlinear regression analysis. * indicates differences from WT controls at *p* < 0.05. ** indicates significant differences between groups at *p* < 0.05.

Previous studies have shown that C57BL/6J mice were relatively resistant to denudation-induced neointima formation in the carotid arteries compared to other inbred mouse strains [14], but the absence of apoER2 expression in C57BL/6J mice (*Lrp8^−/−^* mice) resulted in robust injury-induced neointima with extensive fibrosis [9]. The current study examined neointima in the carotid arteries of wild-type, *Lrp8^+exon19^* and *Lrp8^Δexon19^* mice 14 days after endothelial denudation injury to determine if exon 19 cytoplasmic insert of apoER2 is necessary to protect against this injury-induced vascular occlusive disease. The results showed that whereas the neointima area in the injured carotid arteries of *Lrp8^Δexon19^* mice was slightly, albeit not significantly, increased compared to that observed in wild-type mice, the neointimal area in *Lrp8^+exon19^* mice was significantly less than that observed in wild-type and *Lrp8^Δexon19^* mice (Figure 1a,b).

In view of our previous studies showing that the robust fibrotic neointima observed in *Lrp8^−/−^* mice was due to impaired cytokinesis and the accelerated senescence of vascular smooth muscle cells [9], additional experiments were performed to assess growth rates of smooth muscle cells isolated from the aortas of wild-type, *Lrp8^+exon19^* and *Lrp8^Δexon19^* mice. The expression of either apoER2 exon 19 variant was sufficient to prevent accelerated smooth muscle cell senescence. Interestingly, the exclusive expression of the full-length apoER2 variant with exon 19 cytoplasmic insert was found to reduce smooth muscle cell growth compared to wild-type cells that expressed both exon 19 variants (Figure 1c). In contrast, smooth muscle cells that exclusively expressed the apoER2 variant without exon 19 were found to have an increased growth rate compared to wild-type cells (Figure 1c). Since smooth muscle cell growth plays a significant role in neointima expansion after endothelial denudation, the protective effects of the apoER2 variant with exon 19 are likely due at least in part to the slower growth rates of these smooth muscle cells. Taken together, we interpret these data to indicate that the expression of either of the spliced forms of apoER2 is sufficient to modulate smooth muscle cell response in limiting injury-induced neointimal formation and suppress the formation of robust fibrotic neointima, but the cytoplasmic insert encoded by exon 19 is necessary to limit smooth muscle cell growth for complete protection against denudation-induced vascular occlusive disease.

### 3.2. apoER2 Cytoplasmic Splice Variants Have Minimal Impact on Atherosclerosis in Ldlr^−/−^ Mice

**Figure 2:**
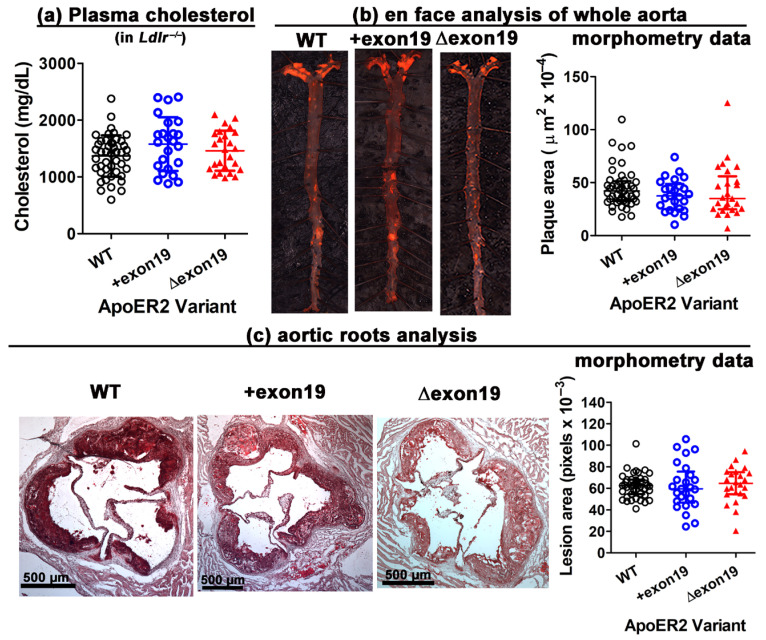
The effect of the apoER2 exon 19 splice variant on atherosclerosis. *Lrp8^+/+^Ldlr^−/−^* (WT, n = 45), *Lrp8^+exon19^Ldlr^−/−^* (+exon19, n = 25) and *Lrp8^Δexon19^Ldlr^−/−^* (Δexon19, n = 25) mice were fed a Western diet for 16 weeks. (**a**) Plasma cholesterol levels were measured after 15 weeks. The data passed the Shapiro–Wilk test for normality, and one-way ANOVA with Student–Newman–Keuls post hoc analysis revealed no difference between groups. The data are presented as mean ± SD. (**b**) Representative images and quantification of Oil Red O-stained atherosclerotic lesions in the whole aorta. (**c**) Representative images and quantification of lesion sizes in the aortic roots. The scale bars represent 500 µm. Morphometric data in panels b and c are presented as the median with interquartile range. One-way ANOVA with the Kruskal–Willis post hoc test revealed no differences between groups.

Wild-type, *Lrp8^+exon19^* and *Lrp8^Δexon19^* mice were also mated with the atherosclerosis-susceptible *Ldlr^−/−^* mice to generate *Lrp8^WT^Ldlr^−/−^, Lrp8^+exon19^Ldlr^−/−^* and *Lrp8^Δexon19^Ldlr^−/−^* mice to assess how apoER2 exon 19 splice variants may influence atherosclerosis progression. The *Lrp8^WT^Ldlr^−/−^, Lrp8^+exon19^Ldlr^−/−^* and *Lrp8^Δexon19^Ldlr^−/−^* mice were fed a Western-type high-fat–high-cholesterol diet for 16 weeks prior to atherosclerosis assessment. Due to the *Ldlr^−/−^* background of these animals, all three groups of mice developed robust hypercholesterolemia but plasma cholesterol levels were comparable regardless of the apoER2 variant expressed (Figure 2a).

En face analysis of the whole aorta by oil red O staining showed no difference in atherosclerotic plaque areas in the aorta among the three groups (Figure 2b), and an additional cross-section analysis of the aortic roots also revealed no difference in lesion area between *Lrp8^WT^Ldlr^−/−^, Lrp8^+exon19^Ldlr^−/−^* and *Lrp8^Δexon19^* mice (Figure 2c). These results are in striking contrast with the accelerated atherosclerosis progression to complex lesions observed previously in apoER2-deficient *Lrp8^−/−^Ldlr^−^^/−^* mice [10]. Taken together, these results indicate that the expression of apoER2, regardless of the presence or absence of exon 19 cytoplasmic insert, has no impact on the early phase of atherogenesis with foam cell formation and is sufficient to limit atherosclerosis progression to the more advanced necrotic phase in hypercholesterolemic mice.

### 3.3. Influence of ApoER2 Exon 19 on Body Weight and Adiposity

**Figure 3:**
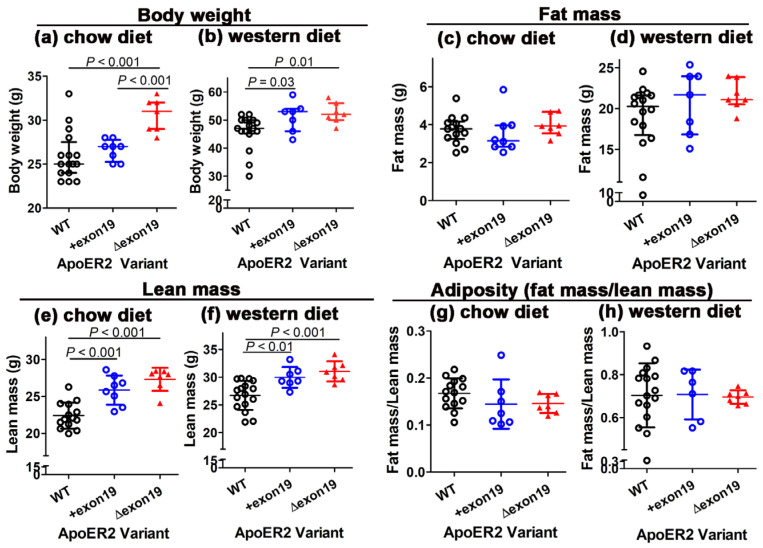
The effect of the apoER2 exon 19 splice variant on body weight, fat and lean mass, adiposity and tissue weight. A total of 16 wild-type (WT), 8 *Lrp8^+exon19^* and 7 *Lrp8^Δexon19^* mice were fed either a basal chow diet or a Western diet for 16 weeks. The data show the body weight of mice on a chow diet (**a**) or a Western diet (**b**), the fat mass of mice on a chow diet (**c**) or a Western diet (**d**), the lean mass of mice on a chow diet (**e**) or a Western diet (**f**) and adiposity as determined by the fat/lean mass ratio of mice fed a chow diet (**g**) or a Western diet (**h**). Data for body weight and fat mass (panels (**a**–**d**)) are presented as the median with interquartile range and were evaluated by one-way ANOVA with the Kruskal–Wallis post hoc test. Data for lean mass and adiposity (panels (**e**–**h**)) are presented as mean ± SD and were evaluated by one-way ANOVA with Student–Newman–Keuls post hoc analysis. Differences between groups are indicated by *p* values, as shown.

**Figure 4:**
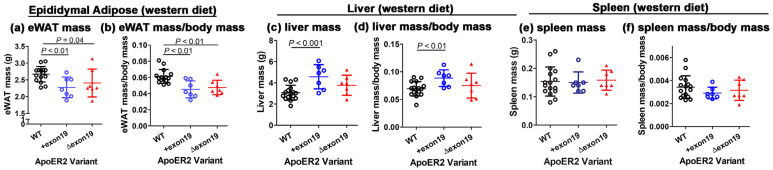
The effect of the apoER2 exon 19 splice variant on tissue weights. The data show the epididymal adipose tissue (eWAT) mass (**a**) and eWAT mass/body mass (**b**), liver mass (**c**) and liver mass/body mass (**d**) and spleen mass (**e**) and spleen mass/body mass (**f**) of wild-type (n = 16), *Lrp8^+exon19^* (n = 8) and *Lrp8^Δexon19^* (n = 7) mice after feeding the Western diet for 16 weeks. All the data satisfied the normality test and were analyzed by one-way ANOVA with the Student–Newman–Keuls post hoc test. Data are presented as mean ± SD with differences between groups indicated by *p* values, as shown.

A previous study showed that apoER2-deficient *Lrp8^−/−^* mice weighed less than their C57BL/6J wild-type counterpart when fed a normal chow diet and displayed less body fat when fed either a normal chow or a Western-type high-fat–high-cholesterol diet [11]. Therefore, the *Lrp8^+exon19^* and *Lrp8^Δexon19^* mice, along with their C57BL/6J wild-type counterparts, were fed either a basal chow diet or a Western-type high-fat–high-cholesterol diet to ascertain the influence of apoER2 exon 19 on body weight and adiposity. Surprisingly, the *Lrp8^Δexon19^* mice exhibited higher body weight compared to wild-type and *Lrp8^+exon19^* mice even under basal chow diet conditions (Figure 3a). Upon feeding the Western-type high-fat–high-cholesterol diet for 15 weeks, both *Lrp8^+exon19^* and *Lrp8^Δexon19^* mice gained more weight than wild-type mice, and *Lrp8^+exon19^* and *Lrp8^Δexon19^* mice no longer showed body weight differences under Western diet conditions (Figure 3b). The additional characterization of body composition revealed no significant differences in fat mass between wild-type, *Lrp8^+exon19^* and *Lrp8^Δexon19^* mice when fed either a basal chow diet or a Western diet (Figure 3c,d). The lower body weight observed in wild-type mice compared to *Lrp8^+exon19^* and *Lrp8^Δexon19^* mice was due to lower lean mass observed in wild-type mice (Figure 3e,f). Accordingly, there was no difference in adiposity, assessed as fat mass/lean mass, between all three groups of mice (Figure 3g,h). Interestingly, despite comparable adiposity among the three groups, epididymal adipose tissue weights were significantly lower in *Lrp8^+exon19^* and *Lrp8^Δexon19^* mice compared to wild-type mice in response to Western diet feeding (Figure 4a,b). Moreover, Western diet-fed *Lrp8^+exon19^* mice, but not *Lrp8^Δexon19^* mice, also displayed increased liver mass (Figure 4c,d). The differences in epididymal adipose and liver weights were organ-specific, as spleen mass was comparable between Western diet-fed wild-type, *Lrp8^+exon19^* and *Lrp8^Δexon19^* mice (Figure 4e,f). Taken together, these data indicate that exon 19 in apoER2 influences body weight in a dietary- and tissue-specific manner via differences in nutrient partitioning among the various tissues. Specifically, the exclusive expression of apoER2 with exon 19 appeared to favor nutrient partitioning to the liver instead of visceral adipose tissues, whereas the exclusive expression of apoER2 without exon 19 may favor nutrient utilization for the growth of lean mass. Consistent with the latter possibility is the report that apoER2 participates in bone development by activating signaling pathways that promote osteoblast differentiation and mineralization [15].

### 3.4. Influence of apoER2 Exon 19 on Adipose Tissue Inflammation

**Figure 5:**
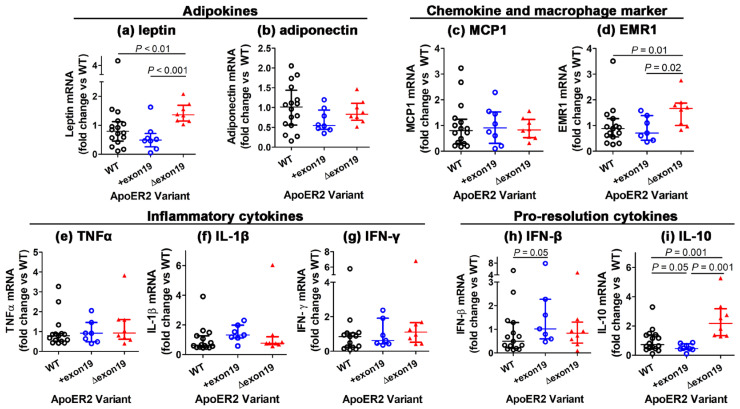
The effect of the apoER2 exon 19 splice variant on adipose tissue gene expression. Total RNA was prepared from the epididymal adipose tissues of Western diet-fed wild-type (WT, n = 15), *Lrp8^=exon19^* (n = 7) and *Lrp8^Δexon19^* (n = 8) mice to analyze the expression of the adipokine genes leptin (**a**) and adiponectin (**b**); the chemokine MCP1 (**c**) and macrophage marker EMR1 (**d**); inflammatory cytokines TNFα (**e**), IL-1β (**f**) and IFN-γ (**g**); and the pro-resolution cytokines IFN-β (**h**) and IL-10 (**i**). The data were evaluated by one-way ANOVA with Kruskal–Wallis analysis and are presented as the median with interquartile range. Significant differences between groups are indicated by *p* values, as shown.

Despite comparable amounts of adiposity in wild-type, *Lrp8^+exon19^* and *Lrp8^Δexon19^* mice, gene expression analysis of epididymal adipose tissues revealed significantly higher levels of adipocyte inflammation in *Lrp8^Δexon19^* mice. Specifically, the expression level of the pro-inflammatory adipokine leptin was found to be elevated in *Lrp8^Δexon19^* mice compared to wild-type and *Lrp8^+exon19^* mice (Figure 5a), whereas comparable expression levels of the anti-inflammatory adiponectin were observed regardless of the apoER2 variant being expressed (Figure 5b). Furthermore, although MCP1 expression levels were similar among all three groups (Figure 5c), there was over-abundance of macrophages in the adipose tissues of *Lrp8^Δexon19^* mice, as evident by the higher expression level of EMR1 that encodes the macrophage-specific marker F4/80 (Figure 5d). Interestingly, the increased macrophage presence did not alter expression levels of inflammatory cytokines such as TNFα, IL-1β and IFN-γ in adipose tissues (Figure 5e–g). In contrast, expression levels of the pro-resolution phase cytokine IFN-β were found to be higher in the adipose tissues of *Lrp8^+exon19^* (Figure 5h), whereas expression levels of IL-10, a cytokine with both pro- and anti-inflammatory properties [16,17], were higher in *Lrp8^Δexon19^* mice (Figure 5i). Collectively, these observations are consistent with the interpretation that the exclusive expression of the apoER2 variant without exon 19 increases adipocyte inflammation with increased macrophage deposition in the adipose tissues. Moreover, the exclusive expression of either forms of apoER2 is sufficient in remodeling macrophage subsets in the adipose tissues to modulate the adipose tissue inflammatory response.

### 3.5. Distinct Effects of apoER2 Exon 19 Splice Variants on Diet-Induced Hyperglycemia and Glucose Intolerance

**Figure 6:**
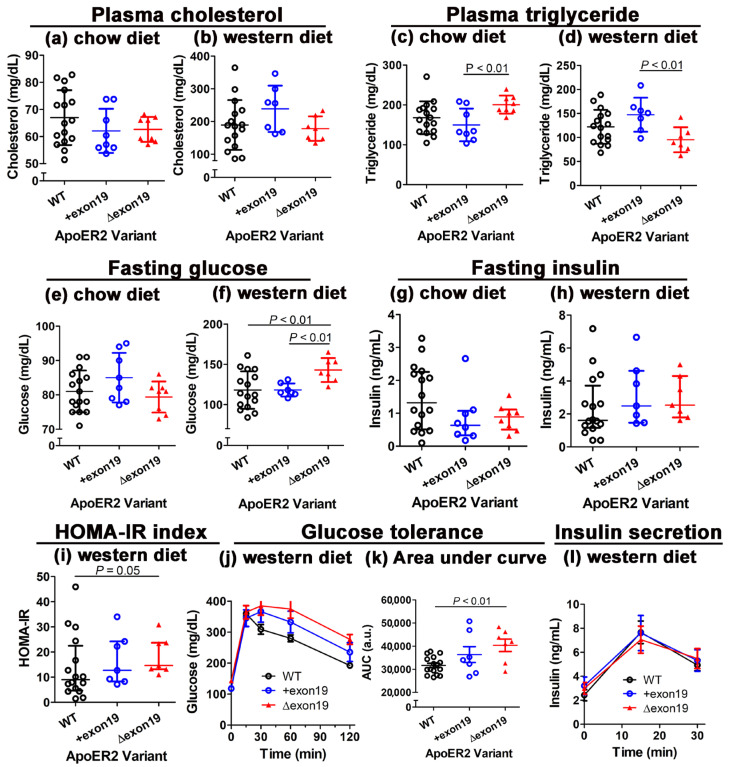
The effect of the apoER2 exon 19 splice variant on plasma lipid, glucose and insulin levels, glucose tolerance and insulin secretion. Blood was collected from wild-type (WT, n = 16), *Lrp8^+exon19^* (n = 8) and *Lrp8^Δexon19^* (n = 8) mice. The data show plasma cholesterol levels of mice on a chow diet (**a**) or a Western diet (**b**), plasma triglyceride levels of mice on a chow diet (**c**) or a Western diet (**d**), blood glucose levels on mice on a chow diet (**e**) or a Western diet (**f**), plasma insulin levels of mice on a chow diet (**g**) or a Western diet (**h**), insulin resistance of mice fed a Western diet (**i**), a glucose tolerance test of Western diet-fed mice after being fed a bolus glucose meal (**j**), an area under the curve analysis of a glucose tolerance test (**k**) and insulin secretion into plasma 15 and 30 min after glucose feeding (**l**). The data were assessed by the Shapiro–Wallis test for normality. All data showing normal distribution (panels (**a**–**f**,**k**) were evaluated by one-way ANOVA with Student–Newman–Keuls post hoc analysis for significance and are presented as mean ± SD. Data that did not show normal distribution (panels, (**g**–**i**)) were analyzed by the Kruskal–Wallis test, followed by Dunn’s test, and are presented as the median with interquartile range. Differences at *p* < 0.05 were considered significant differences between groups, as shown.

Elevated visceral adipose tissue inflammation with increased leptin production observed in Western diet-fed *Lrp8^Δexon19^* mice suggested that the exclusive expression of the apoER2 variant without exon 19 may accelerate cardiometabolic diseases [18,19,20]. This hypothesis was examined by comparing plasma lipids, glucose and insulin levels in wild-type, *Lrp8^+exon19^* and *Lrp8^Δexon19^* mice under a basal chow diet and after 15 weeks of feeding a Western-type diet. While no apoER2 variant-specific differences in plasma cholesterol levels were observed under either dietary condition (Figure 6a,b), plasma triglyceride levels were elevated in chow-fed *Lrp8^Δexon19^* mice but were lower in Western diet-fed *Lrp8^Δexon19^* mice (Figure 6c,d). The lower plasma triglyceride levels observed in all three groups of Western diet-fed mice compared to chow-fed animals are consistent with the phenotype of C57BL/6J mice [21]. Regardless, the minimal differences in plasma lipid levels indicated that the apoER2 cytoplasmic splice variants have minimal effect on plasma lipoprotein metabolism. More importantly, while fasting plasma glucose levels were comparable among chow-fed wild-type, *Lrp8^+exon19^* and *Lrp8^Δexon19^* mice (Figure 6e), fasting glucose levels were significantly elevated in Western diet-fed *Lrp8^Δexon19^* mice (Figure 6f). In contrast, fasting insulin levels were found to be comparable among all three groups of mice under both chow and Western diet conditions (Figure 6g,h). The specific elevation of plasma glucose levels in Western diet-fed *Lrp8^Δexon19^* mice resulted in higher insulin resistance, as estimated by homeostatic model assessment for insulin resistance (HOMA-IR) (Figure 6i). The increased glucose intolerance observed in Western diet-fed *Lrp8^Δexon19^* mice was confirmed by an oral glucose tolerance test that showed delayed glucose clearance in these animals compared to Western diet-fed wild-type and *Lrp8^+exon19^* mice (Figure 6j,k). Consistent with results showing no difference in plasma insulin levels, the diet-induced glucose intolerance observed in *Lrp8^Δexon19^* mice was not due to impaired insulin secretion (Figure 6l). Taken together, these results indicated that the exclusive expression of the apoER2 variant without exon 19 leads to increased sensitivity to diet-induced hyperglycemia due to impaired glucose clearance capacity. Thus, exon 19 of apoER2 plays an important role in limiting diet-induced metabolic diseases.

## 4. Discussion

Previous studies showed accelerated smooth muscle cell senescence in the absence of apoER2, leading to a senescence-associated secretory phenotype that promotes massive fibrotic neointimal formation in response to endothelial denudation-induced arterial injury [9]. The current study showed that accelerated senescence was not observed in smooth muscle cells expressing either of the apoER2 cytoplasmic splice variants. In fact, smooth muscle cells that exclusively expressed the apoER2 variant without exon 19 displayed a faster growth rate compared to wild-type cells that expressed both variants, whereas smooth muscle cells that expressed the apoER2 variant with exon 19 displayed a slower growth rate. Since smooth muscle cell growth and endothelial repair after denudation are key determinants of neointima formation, these differences in growth rate between smooth muscle cells expressing apoER2 with or without exon 19, along with the impaired re-endothelialization after denudation observed with apoER2 exon 19 deletion [22], resulted in a slight but statistically significant increase in neointima size after the endothelial denudation of the carotid arteries in *Lrp8^Δexon19^* mice compared to that observed in *Lrp8^+exon19^* mice, whereas the neointima in the denuded arteries of *Lrp8^+exon19^* mice were smaller than that observed in wild-type mice that expressed both apoER2 splice variants. The variant-specific differences in smooth muscle cell growth and neointima expansion are likely due to the interaction between the proline-rich domain in exon 19 and the adaptor protein SHC1 and the subsequent recruitment and activation of PP2A to dampen Akt activation to suppress cell growth [4]. Thus, the absence of apoER2 exon 19 in cells may result in enhanced cell growth and neointima formation. Conversely, the exclusive expression of the full-length apoER2 variant with the exon 19 cytoplasmic insert may suppress cell growth and limit neointima formation compared to wild-type cells that express both apoER2 splice variants.

ApoER2 deficiency has been shown to have limited impact on the initial phase of diet-induced atherogenesis, but it accelerates the advancement of atherosclerotic lesions to a complex phenotype with extensive necrosis [10]. The current study revealed that despite the differences in smooth muscle cell growth and denudation-induced neointima expansion between wild-type, *Lrp8^+exon19^* and *Lrp8^Δexon19^* mice, Western diet-induced atherogenesis in hypercholesterolemic mice did not display apoER2 exon 19 splice variant preferences. Moreover, minimal necrosis was observed in the lesion areas of these mice. Based on the results of this study and the previous study, the accelerated progression of atherosclerotic lesions to the necrotic phase with apoER2 deficiency is likely due to the impairment of cytokinesis and premature smooth muscle senescence and cell death. Thus, consistent with results of smooth muscle cell growth and neointima expansion, the expression of apoER2 regardless of the presence or absence of exon 19 is sufficient to suppress atherosclerotic lesion advancement to a more complex lesion with significant necrosis. One difference between the apoER2 variant-specific difference in injury-induced neointima expansion and the lack of variant preference in atherosclerosis lesion sizes may be due to the importance of smooth muscle cell growth and endothelial repair after denudation injury in neointima formation, as well as its lesser role in diet-induced atherosclerosis, which is predominantly a lipid- and inflammation-driven cardiovascular disease caused by monocyte infiltration and foam cell formation in the vessel wall. Hence, the similarities in atherosclerotic lesion size observed in wild-type, *Lrp8^+exon19^* and *Lrp8^Δexon19^* mice indicated that apoER2 exon 19 splice variants have limited influence in macrophage lipid accumulation and foam cell formation. Moreover, the differences between the influence of the apoER2 variants on injury-induced neointima formation and diet-induced atherosclerosis re-emphasize that the two vascular occlusive diseases are modulated by distinct mechanisms and genetic determinants [14].

In addition to modulating sensitivity to diet-induced atherogenesis and atherosclerotic lesion progression, apoER2 has been shown to play a role in modulating diet-induced changes in body weight, adiposity, hyperglycemia and hyperinsulinemia. In particular, global apoER2 deficiency reduces body weight and adiposity but accelerates hyperglycemia onset due to impaired glucose-induced insulin secretion [11]. In contrast, bone marrow-specific apoER2 deficiency, presumably due to the defective expression of apoER2 in myeloid cells, was found to be more sensitive to diet-induced adiposity and hyperinsulinemia compared to wild-type mice [11]. Additionally, apoER2-deficient macrophages exhibited inflammation resolution impairment with reduced secretion of IFN-β and IL-10 in response to toll-like receptor 4 activation [11]. The current study revealed no difference in adiposity between wild-type, *Lrp8^+exon19^* and *Lrp8^Δexon19^* mice under either chow- or Western diet-fed conditions, thus indicating that the expression of either apoER2 variant is sufficient and the exon 19 motif is not necessary for adiposity maintenance. However, the apoER2 splice variants influence nutrient partitioning in response to Western diet feeding. Specifically, exclusive expression of the apoER2 with exon 19 variant reduces nutrient partitioning to epididymal adipose tissue with a corresponding increase in partitioning to the liver. The cell type by which apoER2 exon 19 variant expression alters nutrient partitioning between epididymal adipose tissues and liver remains unclear. However, it is of note that apoER2 is not expressed in either adipocytes or hepatocytes [23], thus indicating an indirect effect by which apoER2 exon 19 splice variants expressed in other cell types may be involved. Since apoER2 is highly expressed in neurons and endothelial cells [23], both of which play key roles in peripheral nutrient partitioning [24,25], it is possible that apoER2 exon 19 splice variant expression in these latter cell types may play an important role in dictating nutrient partitioning between adipose tissues and the liver. Additional experiments are necessary to test this possibility.

In contrast to the effects of apoER2 exon 19 variant expression on nutrient partitioning, the exclusive expression of apoER2 without exon 19 increases body weight and lean mass. Since apoER2 deficiency has been shown to reduce osteoblast differentiation and mineralization [15], we interpret the *Lrp8^Δexon19^* phenotype to indicate that the lack of exon 19 enhances bone formation and matrix mineralization, possibly due to the interaction of this cytoplasmic domain with PP2A to regulate Akt activation that is necessary for osteoblastogenesis [26]. However, the specific cell type by which specific apoER2 exon 19 variants may impact body weight and lean mass need to be confirmed by additional studies.

Western diet-fed *Lrp8^Δexon19^* mice also exhibited increased adipocyte inflammation, as characterized by increased expression of leptin, as well as the increased presence of macrophages, as characterized by higher EMR1 mRNA levels, compared to their wild-type and *Lrp8^+exon19^* counterparts. Interestingly, despite the increased presence of F4/80 macrophages in the adipose tissues, the expression of the macrophage-specific inflammatory cytokine TNFα, as well as other pro-inflammatory cytokines such as IL-1β and IFN-γ in *Lrp8^Δexon19^* mice, was comparable to that observed in the adipose tissues of wild-type and *Lrp8^+exon19^* mice. Thus, adipose tissue inflammatory response was not dependent on the specific apoER2 exon 19 splice variant expressed in macrophages. Moreover, the expression of IL-10, a cytokine with both pro- and anti-inflammatory properties, including the facilitation of inflammation resolution, was higher than that observed in wild-type and *Lrp8^+exon19^* mice, while the expression of another inflammation resolution cytokine, IFN-β, was not affected in *Lrp8^Δexon19^* adipose tissues. In contrast, IFN-β expression was found to be higher in adipose tissues of *Lrp8^+exon19^* mice. The latter result is opposite to that observed with macrophages isolated from *Lrp8^−/−^* mice, which showed reduced expression of inflammation resolution cytokines [11], but is in agreement with studies demonstrating that IFN-β overexpression attenuates adipose tissue inflammation and diet-induced obesity and maintains glucose homeostasis [27]. We interpret these collective data to indicate that the apoER2 exon 19 motif is not required in macrophages for the initial phase of inflammatory response but is required to mediate inflammation resolution, and, hence, the increased IL-10 expression observed in adipose tissues of *Lrp8^Δexon19^* mice is not a direct effect of apoER2 in macrophages. Since IL-10 is synthesized primarily by T lymphocytes, it is possible that the increased IL-10 observed in *Lrp8^Δexon19^* is derived from lymphocytes recruited to the adipose tissues in response to elevated adipocyte inflammation to tamper tissue inflammation. The lack of elevated inflammatory cytokine production, despite the increased macrophage presence in the adipose tissues of *Lrp8^Δexon19^* mice, is consistent with this hypothesis. Although the cell type responsible for the differences in inflammatory and anti-inflammatory cytokine production in adipose tissues of wild-type, *Lrp8^+exon19^* and *Lrp8^Δexon19^* mice remains unidentified and requires additional experimentation, our data clearly indicate that the two apoER2 splice variants have different effects on adipose tissue remodeling that cause differential inflammatory responses. Moreover, how the Δexon19 variant of apoER2 elevates leptin expression in adipocytes, which do not express apoER2, remains unknown. It is possible that neuronal dysfunction in the hypothalamus and/or endothelial dysfunction with reduced nitric oxide synthase-3 activity [22] are responsible for increased adipocyte leptin expression in *Lrp8^Δexon19^* mice. These possibilities are worthy of additional experiments in future studies for clarification.

The results of the current study also showed comparable glucose and insulin levels, as well as glucose tolerance, between Western diet-fed wild-type and *Lrp8^+exon19^* mice, but elevated hyperglycemia and insulin resistance with glucose intolerance were observed in *Lrp8^Δexon19^* mice. The distinct metabolic phenotypes of *Lrp8^+exon19^* and *Lrp8^Δexon19^* mice are in striking contrast to the metabolic phenotype of global *Lrp8^−/−^* mice, which displayed hyperglycemia but not hyperinsulinemia due to impaired insulin secretion, or bone marrow-specific apoER2-deficient mice, which exhibited hyperinsulinemia and hyperglycemia due to exacerbated tissue inflammation and inflammation resolution defects [11]. The current study showed that insulin secretion was not impaired in *Lrp8^+exon19^* and *Lrp8^Δexon19^* mice, thus indicating that the expression of either apoER2 splice variant is sufficient to mediate insulin secretion. However, the *Lrp8^Δexon19^* mice displayed exaggerated insulin resistance and glucose intolerance in response to Western diet feeding, thus indicating that apoER2 exon 19 is necessary to prevent accelerated diabetes onset. Since the *Lrp8^Δexon19^* mice did not show inflammation resolution impairment, the accelerated diabetes onset observed in these animals was likely due to a selective increase in leptin synthesis [20]. This hypothesis needs to be confirmed in future experiments.

## 5. Conclusions

In conclusion, this study revealed distinct roles of each apoER2 exon 19 splice variant in modulating cardiovascular and metabolic diseases. In terms of cardiovascular occlusive diseases, the results showed that the expression of either splice variant is sufficient to impede denudation-induced neointima fibrosis and diet-induced atherosclerotic lesion advancement to a necrotic phenotype. However, the expression of the apoER2 splice variant containing exon 19 is necessary for complete protection, whereas the splice variant without exon 19 is not sufficient for the complete suppression of neointima formation due to their differential effects on the rate of smooth muscle cell growth. Moreover, the expression of either splice variant is sufficient to limit hypercholesterolemia-induced atherosclerotic lesion advancement to the necrotic phase, and neither splice variant has an influence on the early phase of atherogenesis with foam cell formation and lipid deposition in the vessel wall. In terms of diet-induced metabolic diseases, the expression of either apoER2 splice variant is necessary and sufficient to mediate insulin secretion, but the expression of the exon 19 cytoplasmic insert is necessary to suppress adipocyte inflammation and protect against diet-induced insulin resistance and glucose intolerance. Taken together, these results indicate that the apoER2 cytoplasmic variants have distinct and cell type-specific roles in influencing the development of cardiometabolic diseases. However, a limitation of this study is the focus on the specific functions and roles of each apoER2 exon 19 splice variant in cardiometabolic disease modulation. Further studies are necessary to identify the factors that may drive apoER2 exon 19 splicing and whether these factors are regulated in response to exogenous stimuli, such as diet and metabolic stress. These additional studies may lead to additional insights that can be leveraged to lower cardiometabolic disease risk.

## Figures and Tables

**Table 1:** Primer sequences used for qPCR analysis.

Name	Forward Primer	Reverse Primer
Cyclophilin	TCATGTGCCAGGGTGGTGAC	CCATTCAGTCTTGGCAGTGC
Leptin	GCAGCACACGATGGAAGCACTTAT	TTGGGCAGACCCATCAATAGGATT
Adiponectin	AAAGATGTGAAGGTGAGCCTCTTC	CTGGTCCACATTCTTTTCCTGAT
MCP1	CCTCCTCCACCACCATGCA	CCAGCCGGCAACTGTGA
EMR1	TGTCTGACAATTGGGATCTGCCCT	ATACGTTCCGAGAGTGTTGTGGCA
TNFα	ATCCGCGACGTGGAACTG	ACCGCCTGGAGTTCTGGAA
IL-1β	CTACAGGCTCCGAGATGAACAAC	TCCATTGAGGTGGAGAGCTTTC
IFN-γ	CTACACACTGCATCTTGGCTTTG	TGACTGTGCCGTGGCAGTA
IFN-β	CCATCATGAACAACAGGTGGAT	GAGAGGGCTGTGGTGGAGAA
IL-10	TGAATTCCCTGGGTGAGAAGCTGC	TGGCCTTGTAGACACCTTGGTCTT

## Data Availability

All supporting data are available within the article and are available from the corresponding author upon reasonable request.

## Abbreviations

The following abbreviations are used in this manuscript:

ApoER2	Apolipoprotein E receptor-2
LDL	Low-density lipoproteins
PP2A	Protein phosphatase-2A
JNK	cJun N-terminal kinase
JIP	JNK-interacting protein
LDLR	LDL receptor
PCR	Polymerase chain reaction
WT	Wild type
ANOVA	Analysis of variance
MCP1	Monocyte chemoattractant protein-1
EMR1	EGF-like module receptor 1 (also called F4/80)
TNFα	Tumor necrosis factor-α
IL	Interleukin
IFN	Interferon
HOMA-IR	Homeostatic model assessment for insulin resistance
Akt	Akt serine/threonine kinase (also called protein kinase B)
